# Hard to Please

**Published:** 1874-07-15

**Authors:** 


### Editorial Department.

### HARD TO PLEASE.

TO a liberal, earnest mind, accustomed to work, and manfully striving to make the world better, few things appear more contemptible than a *chronic fault-finder*, or a self-complacent *Pecksniff*. The leading editorial in the Philadelphia *Medical Times*, for June 27th, forcibly reminds us of the union of both these characters in one person. The article relates to the American Medical Association, which it derisively calls the "*redoubtable* organization." And after alluding to what the writer calls "remarkable statistics," presented by Dr. Sayre, of New York, in the Surgical Section, he continues: "Leaving out of sight this matter, the Detroit gathering does not appear to have yielded any scientific fruits whatever." Of course not. There were only eight or ten well-written papers presented and discussed in the Section on Practice of Medicine, Materia Medica, and Physiology; as many in the Section on State Medicine and Public Hygiene; half that number in each of the Sections on Obstetrics and Surgery, including such writers and teachers as Gross, of Philadelphia; Sayre and Beard, of New York; Moore, of Rochester; Dunlap, of Ohio; Morris and Howard, of Baltimore; Bell, of Brooklyn; Parvin, of Indianapolis, etc. What a pity that some of these gentlemen had not bethought themselves to have quoted, at least, one line from the editorial department of the Philadelphia *Medical Times*, and thereby given the Detroit meeting some slight aroma of "scientific fruit." The following, however, is the most remarkable paragraph in the editorial of the *Times*: "For the sake of *our* foreign contemporaries, *we* want to deny emphatically that the convocation was in any true sense representative of the American profession. We do not in any way wish to disparage our Western brethren, but it is a simple fact that by far the largest portion of the leading minds of the profession are to be found in our Eastern cities. The most influential periodicals, with a very few exceptions, are there issued; the American medical works almost all have such nativity; the chief medical schools of the country are there situated, and the facilities for higher medical self-education, for study and investigation, do there most abound. Yet, at the late meeting, these cities were scarcely represented at all. Boston, we are informed, sent one delegate; New York thirteen, and Philadelphia nine. Moreover, with very few exceptions, these representatives were not men of prominence at home—excellent physicians, no doubt, but not writers, teachers, or practitioners of national reputation."

How kind, how very considerate, this Philadelphia editor is, to let his "foreign contemporaries" know that an association in which the prominent actors were such obscure men ("excellent physicians, no doubt,") as J. M. Toner and J. J. Woodward, of Washington; S. D. Gross and Washington Atlee, of Philadelphia; John Morris and E. L. Howard, of Baltimore; A. N. Talley, of Charleston; J. Marion Sims, A. N. Bell and L. A. Sayre, of New York; E. M. Moore, of Rochester; J. P. White, of Buffalo; J. B. Johnson and A. H. Gregory, of St. Louis; J. A. Murphy, of Cincinnati, and W. K. Bowling and W. T. Briggs, of Nashville, was in no "true sense representative of the American Profession." How thankful "our foreign contemporaries" ought to be, that we have, at least, one editor in America who is kind enough to prevent their being imposed upon! But will not J. B. Lippincott & Co., publishers of the *Medical Times*, please inform us in the next issue of that most valuable journal, *who* its editor is? For we, out in the "*Far West*," are not sure that it would not be best to raise a subscription in the Mississippi Valley, sufficient to pay the traveling expenses and hotel bills of said editor, and induce him to attend the next meeting at Louisville, so that Philadelphia may have a representative worthy of the *Emporium* of Medical Science, and the American Medical Association may present in some small degree a "true representative" character. We have no doubt but the "Western brethren" would subscribe liberally for this purpose; and if the journey should appear to be so "far West" as to frighten the learned gentleman, they would hire some good, motherly lady to accompany and take care of him, especially while crossing the more rugged parts of the Alleghany Mountains.

